# Isthmocele: A Case Report and Review of an Increasingly Common Gynecological Hurdle

**DOI:** 10.7759/cureus.71988

**Published:** 2024-10-21

**Authors:** Annabel Stout, Lucy Dicks-Ilori, Alaa Elghobashy

**Affiliations:** 1 Obstetrics and Gynecology, Worcester Royal Hospital, Birmingham, GBR; 2 Anaesthetics, Sandwell and West Birmingham National Health Services (NHS) Trust, Birmingham, GBR; 3 Gynecologic Oncology, Wolverhampton Royal Hospital, Birmingham, GBR

**Keywords:** caesarean scar defect, isthmocele, isthmocele repair, post-caesarean niche, uterine niche

## Abstract

Isthmocele is emerging as an increasingly common yet troublesome diagnosis. The spectrum of potential symptoms is large, with subsequent management strategies continuing to evolve. For clinicians, having first recognized the inconsistencies in presentation, familiarity with the differing treatment options is important. We reviewed an interesting case whereby a large isthmocele with a secondary pseudocyst was diagnosed. The diagnosis was 10 years following cesarean section and three years following vaginal birth after cesarean section. Complex robotic surgical management was required owing to a large defect in close association with the urinary bladder. The authors provide an essential overview for obstetricians, gynecologists, and general clinicians to translate into practice. Discussion of the important features in the presentation and diagnosis of isthmocele is held. Particular focus is placed on management strategies and essential preoperative considerations for these complicated and variable defects.

## Introduction

Isthmocele, an ‘indentation of the uterine myometrium at the site of the cesarean section scar, with a depth of at least 2 mm’ is an increasingly described phenomenon [1]. Also referred to as a ‘cesarean scar defect/niche’, these pouch-like defects in the uterine isthmus occur in a semicircular or triangular shape [2,3]. Symptoms have been collated together as ‘cesarean scar syndrome’, with presentation incidence soaring in recent years [4,5]. An objective definition is lacking, thus reported prevalence varies hugely according to assessment technique [4]. Undoubtedly, with the increasing proportion of cesarean section deliveries, the prevalence of isthmocele is likely to follow suit. Despite the increasing prevalence, the investigation and management of isthmocele remain poorly understood. Below we outline best practices according to the literature, in the context of an interesting case.

## Case presentation

A 41-year-old woman presented with a three-year history of amenorrhea and associated lower abdominal pain. There was no abnormal bleeding, discharge, or menopausal symptoms. Previously, the patient had undergone three vaginal deliveries, one cesarean, and most recently, a vaginal birth after a cesarean three years prior to presentation. The examination was normal.

**Figure 1:**
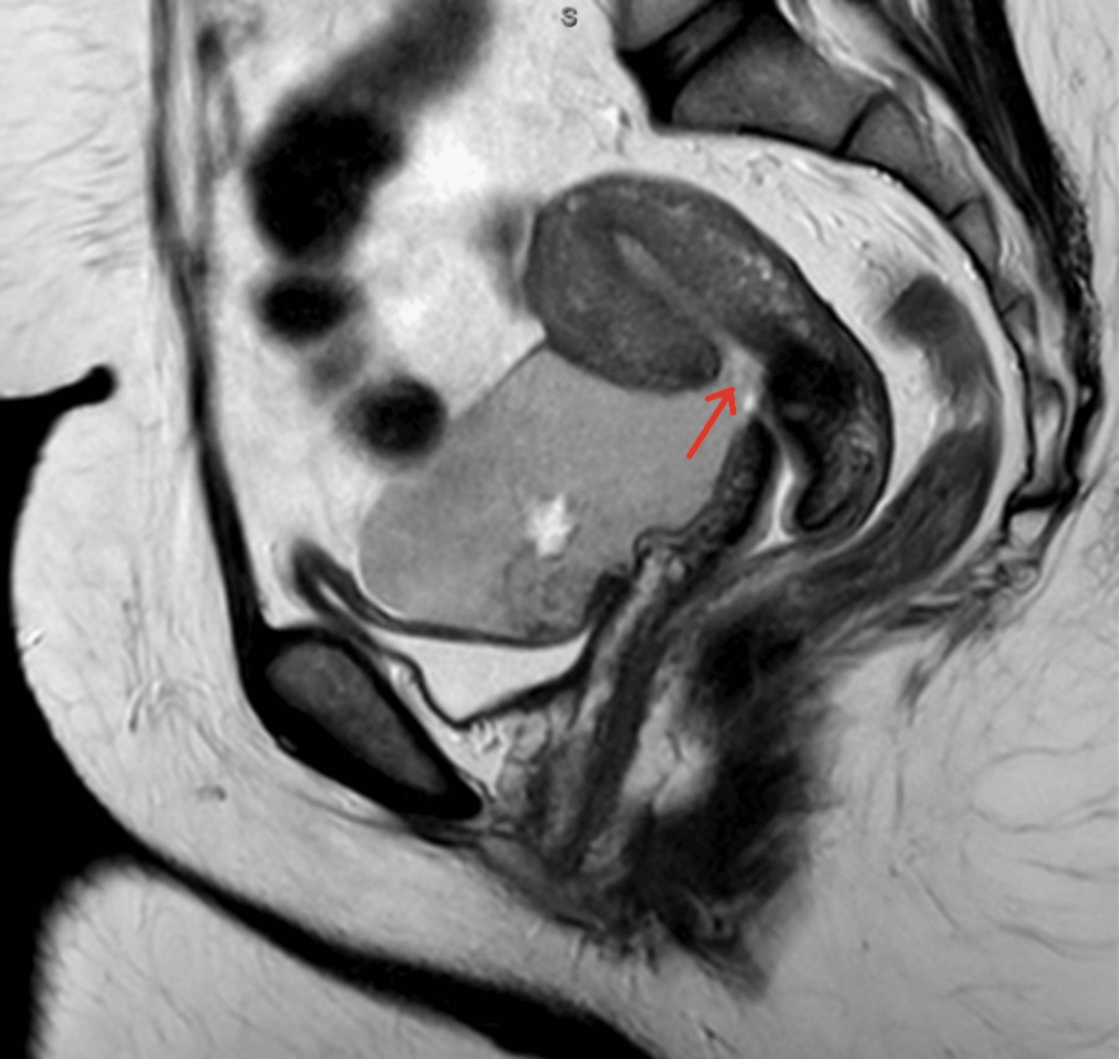
MRI demonstrating the large isthmocele

No abnormalities were seen on routine blood tests. Transvaginal ultrasound revealed a complex cystic mass with echogenic contents situated on the right superior anterior border of the uterus. MRI (magnetic resonance imaging) of the pelvis demonstrated a 6x9x6cm cystic collection anterior to the uterus suggestive of hematoma, communicating with the previous cesarean section scar (Figure 1). A diagnosis of isthmocele, potentially following partial scar rupture, is seen, and accordant management of the cesarean scar niche commenced.

Progesterone was trialed; however, no withdrawal bleed was induced; a referral to a hysteroscopy clinic was arranged. Outpatient hysteroscopy demonstrated a small aperture anterior to the internal cervical os. Entry into this aperture led to the immediate drainage of 100 ml of foul-smelling old blood. Further attempts at hysteroscopy were abandoned due to patient discomfort.

**Figure 2:**
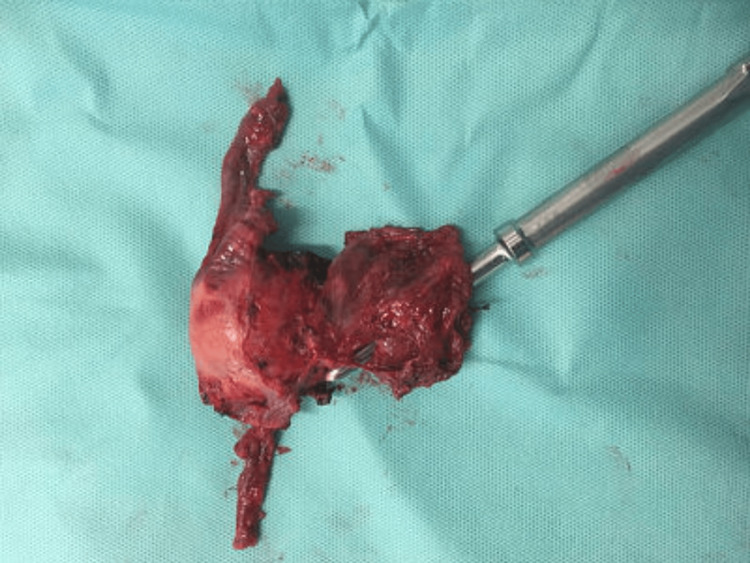
Operative specimen following hysterectomy. Hagar dilator demonstrates position of isthmocele at uterine lower segment

**Figure 3:**
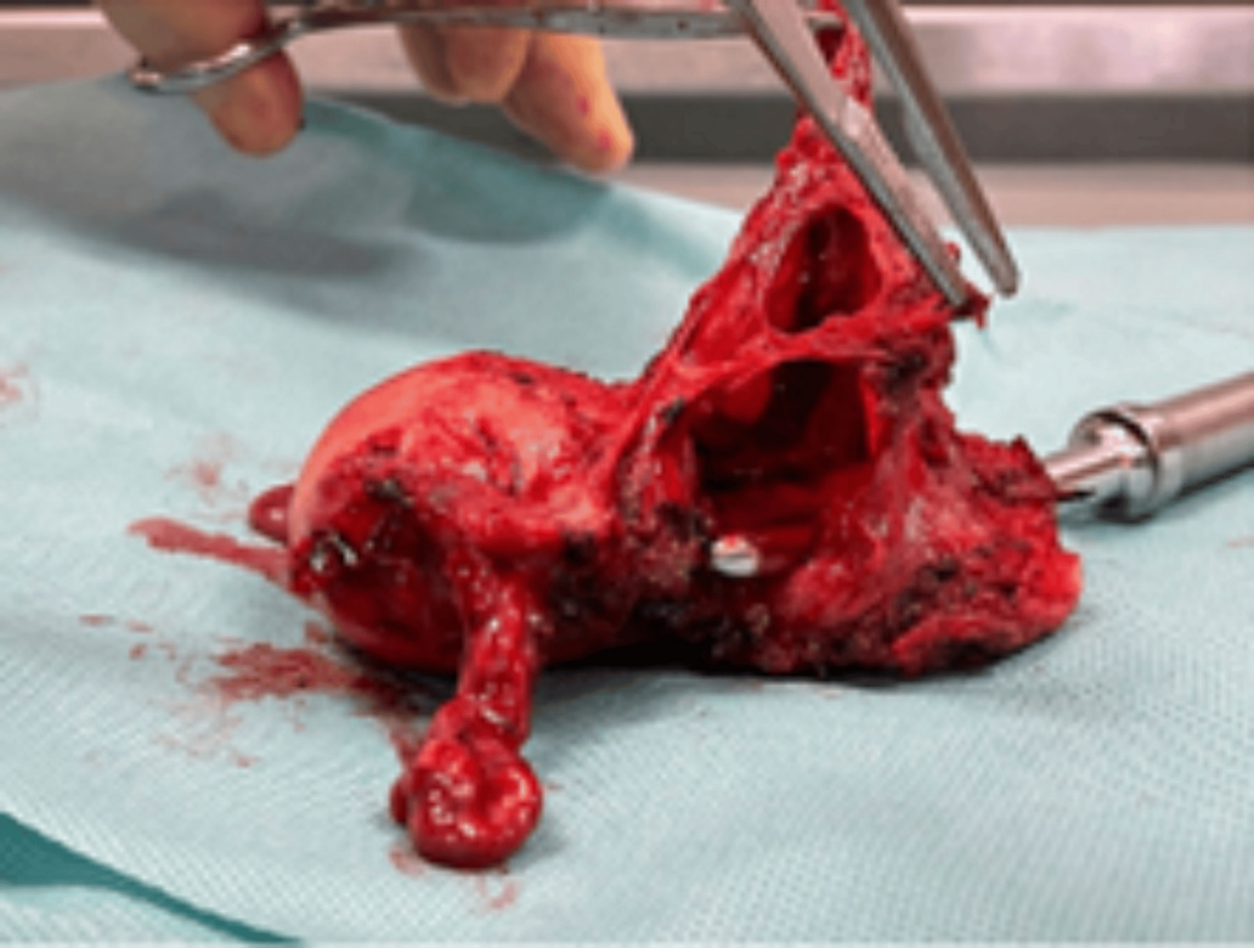
Demonstration of cavity occupying haematomatous collection secondary to isthmocele

The patient had no desire for future fertility and was eager for definitive management. Robotic-assisted total hysterectomy and bilateral salpingectomy with conservation of ovaries were planned. A multidisciplinary approach was adopted, with the involvement of the urology team. At commencement, findings of a large isthmocele were supported by a 9-10 cm cavity filled with yellow fluid between the urinary bladder and anterior uterine wall/cervix. A pseudocyst formed secondary to the collection of menstrual blood draining through the isthmocele was evident. The introduction of the V-care uterine manipulator was performed under laparoscopic guidance to prevent injury; indeed, the manipulator passed immediately into the isthmocele with no suggestive operator feedback. A tubal uterine manipulator was therefore utilized to facilitate surgical dissection. Bilateral ureters were lateralized down to the level of the bladder. Ureteric stenting was considered but not required, as visualization was optimal secondary to their dissection. The pseudocyst was slowly and meticulously separated from the bladder, after which the lower uterine segment was exposed and the hysterectomy completed (Figures 2, 3). 

Histology confirmed deciduosis of the anterior uterine scar with cystic tissue sections with associated chronic inflammation. No issues were reported post-operatively.

## Discussion

Etiology

Isthmocele can present acutely after a cesarean section or be delayed by years, as evidenced in our case. The timing of occurrence vs. presentation remains unclear. The relationship to the closure technique is frequently postulated. Early data has shown a reduced risk of scar defects following double-layer closure [3]. Specifically, the inclusion of decidua, considering separating deep and superficial myometrial closure in cases of thick myometrium, is advised [3]. Necrosis, often brought about by locking sutures, non-perpendicular edges, or a tight second layer closure, is also postulated to increase risk [3]. Operatively, lower uterine incisions and cesarean in prolonged or advanced labor are also risk factors [3]. Retraction-induced defects are noted secondary to anterior abdominal wall adhesions, or retroflexed uteri [6]. The angular orientation of the scar leads to altered anatomy and defective healing. Patient risk factors include a higher BMI, an increasing number of previous cesarean sections, and gestational diabetes [7]. It is important to note that high-quality evidence regarding the cause and subsequent prevention is lacking, with the majority of advice either postulated or based on low-quality evidence only.

Our case raises an interesting new notion regarding etiology, given the likely development following scar dehiscence during vaginal birth after a cesarean, as opposed to following the cesarean itself. Such etiology has not been well described in the literature previously; however, pathophysiologically, it is feasible.

Presentation

**Figure 4:**
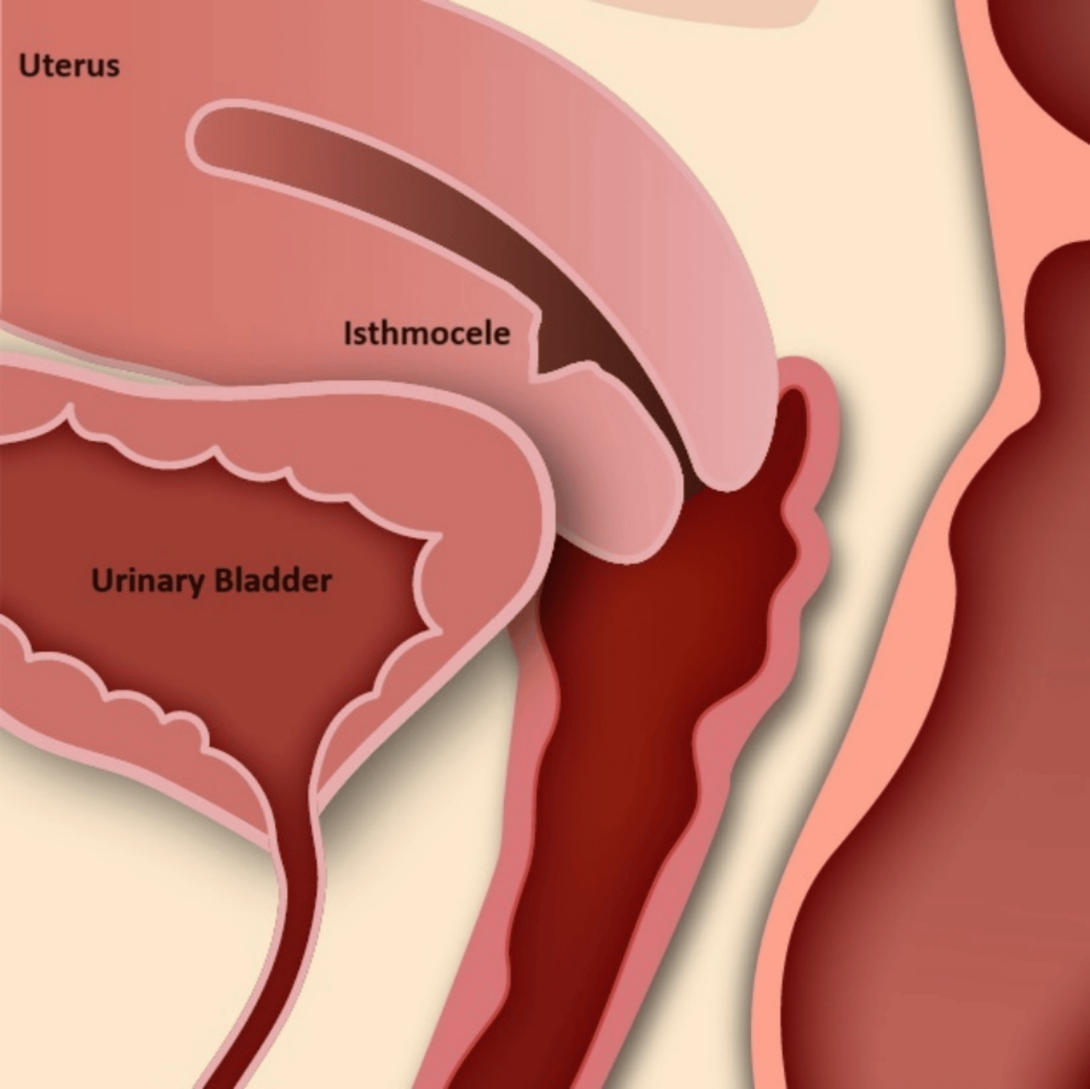
Diagrammatic representation of small isthmocele Image credit: Mark Smith. Permission for use provided

Visualization of a pouch-like niche in the lower uterine segment (Figure 4) aids in understanding common symptoms.

Presentation is routinely related to bleeding anomalies, with menstrual blood pooling in the niche leading to prolonged bleeding, intermenstrual bleeding, or, more rarely, amenorrhea [8]. Studies have reported a positive correlation between defect size and severity of symptoms [8,9]. This is echoed by our case, whereby a large defect led to complete amenorrhea via hematometra. Less commonly, patients can develop secondary pain, either dysmenorrhea, chronic pelvic pain, or infertility [8].

Diagnosis 

Diagnosis can be challenging, not least due to the lack of a standardized definition. Transvaginal ultrasound is a commonly adopted initial investigation for bleeding concerns; however, it is highly operator-dependent. Saline infusion sonohysterogrophy has been shown to demonstrate a higher percentage of defects than ultrasound alone [2,3]. However, access and expertise in this technique are more limited. Hysteroscopic techniques have been described, given their advantage of direct visualization of the size, site, and shape of the lesion. Measurement of remaining myometrial thickness is however not possible hysteroscopically. MRI, an increasingly available and highly accurate imaging technique, is a useful option. Offering improved visualization in challenging cases such as high BMI, MRI also achieves accurate quantification of niche size. MRI can also decipher between blood and other fluid collections, identifying a clear tissue interface, which is helpful in cases such as ours where the bladder lies in close proximity to the scar defect [4].

Management

Management of isthmocele should vary according to patient preference and disease burden. Simple medical management to arrest bleeding has shown symptomatic benefit [10] and should be a primary management strategy in the majority of symptomatic patients. Where medical management is not effective, surgery is considered; however, both the risks and operative challenges should not be underestimated. Where future fertility is not required, hysterectomy is a perfectly reasonable option; surgical heroics are not called for, and familiar procedures should be preferred. Lack of evidence and proven benefit of reparative surgery should be discussed and documented with the patient, particularly where they are seeking fertility. Open, laparoscopic, hysteroscopic, robotic, and vaginal approaches to surgery have been described, with a combination approach also commonly adopted.

Pre-Operative Considerations

Isthmocele repair is surgically challenging. Adequate access, thin tissues, the requirement for good tissue repair post-operatively, loss of tissue planes, and high risk of inadvertent damage represent but a few of the potential complications. Pre-operative multidisciplinary planning is of utmost importance, both between specialists and during the pre-operative multidisciplinary team brief.

Surgical Approach

A hysteroscopic approach aims to resect either one or both isthmocele edges, resolving the defect as opposed to strengthening the residual myometrium. Hysteroscopy is the only approach that does not involve full-thickness resection and primary closure of the defect. Hysteroscopic management is limited, however, by the residual myometrial thickness. Numerous studies excluded patients with residual thickness less than 2-4 mm, and indeed much of the resulting literature warns against hysteroscopic resection in such cases [5,11]. Studies report high success rates in symptom improvement [11,12] and a solely hysteroscopic approach will likely result in improved recovery vs. laparoscopy. Of the utmost importance in such cases is the risk of bladder injury, hence the preference for direct visualization and repair. The relative risk of future childbearing is considered higher in this approach, with a thinner resultant myometrium correlated to an increased risk of uterine rupture [13]. Therefore, although immediate operative complication rates with hysteroscopy are low, the selection of suitable cases is of the utmost importance [11]. Vaginal repair is possible via experienced surgeons, achieving bladder dissection followed by hysterotomy and repair [3]. Such an approach is minimally reported in the literature; however, the majority of narrative reports benefit from a minimal access approach [6,9]. Laparoscopy is a familiar and well-researched technique. Improvement in patient experience vs. laparotomy is well understood, and complex tissue dissection may be aided by the presence of pneumoperitoneum, fine dissection instruments, and utilization of dissecting electrosurgery. Direct repair of a defect is likely to lead to thicker residual myometrial tissue.

Robotic repair, described minimally in the literature, offers numerous advantages. Indeed, La Rosa et al. call for its use to become the gold standard in this area [4]. A robotic approach provides a 3-dimensional image, offering superior dissection with highly mobile instruments, which negates the limitations usually imposed by surgeon dexterity [14]. Such orientation and manipulation of robotic instruments render them of particular use in challenging surgery requiring precise dissection. Cases such as ours have a high risk of inadvertent bladder injury; techniques aiding dissection are therefore of the utmost benefit. Furthermore, robotic surgery has been shown to lead to a reduction in hospital stay and, in some reports, a reduction in postoperative pain, improving the patient experience [15].

Surgical Considerations

Initially, an exact visualization of the defect is essential. The ‘Rendez-Vous Technique’ described by Nirgianakis et al. has been commonly adopted. Here, hysteroscopic and laparoscopic approaches are combined, utilizing the hysteroscopy light to identify the site and estimate the depth of the defect [16]. Alternatively, Api et al. describe the placement of a Hegar dilator vaginally to identify or perforate the site of the niche. This technique is restricted in cases where the bladder lies closely anterior to the defect [17] (Api et al., 2015). In our case, a cup-shaped uterine manipulator provided similar identification.

Following identification of the defect, familiarity with nearby anatomy should be ensured. The bladder is likely to lie in close association with the defect; indeed, the bladder may be involved in full-thickness dehiscence. Early identification of the bladder site should be made during the initial pelvic assessment and continuously referred to during both dissection and repair. Where a high risk of bladder or ureteric injury is suspected, the presence of a urologist in the theater is undoubtedly helpful. Assessment, dissection, and closure of the defect can then be performed. The superiority of a specific suture has not been proven in the literature, and preference is varied. Use of a 3-0 barbed self-retaining suture is a commonly preferred option for laparoscopic suturing due to its ease of use. This fine suture, however, has a low tensile strength, and insurance of a robust closure must be aimed for, particularly in patients planning a future pregnancy. Separately, Setubal et al. recommend a figure of eight sutures in multiple layers. While this may aid the strength of tissue apposition, the risk of blood vessel disruption and subsequent necrosis needs to be considered [18]. Salvador et al. perform a continuous closure in two layers with 0 polyglactin, warning against the creation of excessive tension and subsequent ischemia risk [19]. Aimi et al. closed with a single layer of interrupted 2-0 polyglactin, wishing to reduce the risk of tissue ischemia as their reason for single-layer repair [20]. Optimal suture type and technique remain unknown, and higher-quality evidence in this area is called for.

Table 1 outlines the important perioperative considerations.

**Table 1:** Surgical considerations in isthmocele repair

Operative subject	Surgical options	Considerations to bear in mind	References
Access	Hysteroscopic (inside out repair) Minimal access or vaginal surgery (outside in)	What is the pre-operative myometrial tissue thickness? Where do the operator expertise lie? Does the patient desire fertility in the future?	[5,11,13]
Visualization	Rendez-Vous Technique Hegar dilator Uterine manipulator Hysteroscope alone	What is the defect size? What is the defect shape? Where does the defect lie? What is the relation of the defect to surrounding structures?	[16, 17]
Bladder relation	Position and size of defect Proximity or communication	How does the defect relate to the ureters? How does the defect relation to the bladder trigone? How clear are the tissue planes?	[16, 17]
Instrument choice	Considerate use of electrosurgery	What is the quality of myometrium surrounding the defect and how can this be optimized?	[4,18,19,20]
Closure	Single vs double layer Interrupted vs continuous Delayed vs absorbable	What is the tissue quality? Is there a desire for future fertility? What is the operators preference?	[18,19,20]

## Conclusions

Our case reports a late presentation of a large complex isthmocele where a robotic-assisted hysterectomy was performed as a definitive treatment option. Whilst minimally reported in the literature, the robotic approach offers excellent visualization and a 3D approach for these challenging dissections and enables safe and precise dissection of a challenging defect. Reporting of reparative techniques and results is lacking, despite the increasing prevalence of isthmocele presentation. Documentation of success in resolving both the defect itself and patient symptoms is encouraged.
